# Swan’s Notation

**Published:** 1884-06

**Authors:** Samuel Swan


### SWAN'S NOTATION.

Mr. Editor:—In response to my request to be shown if I was in error in my paper entitled "The Reason of the Faith that is in Me" my friend, Dr. Deschere, of this city, informs me that Hempel's translation of *Chronic Diseases*, so far as my quotation concerning the trituration of Mercury, is not correct. He says the correct translation is, "one grain of purest liquid Quicksilver has to be triturated with three times one hundred grains of sugar of milk for over three hours to have it one million times diluted in powders, etc., * * * as is taught at the end of this part, with regard to developing the power of other drug medicinal substances." The first paragraph without the last might be construed to mean that the trituration was to be made in accordance with my inference from Hempel's translation. Assuming that Dr. Deschere's translation is correct (and as he is a ripe scholar I have no reason to doubt it), the idea that my notation of potencies is sanctioned by Hahnemann is dissipated.

But my assertion still holds true, that the peculiar mode of potentizing medicines gives no clue to their therapeutic effect, that knowledge being only acquired by experience.

SAMUEL SWAN.

